# Hyperactivation of the habenula as a link between depression and sleep disturbance

**DOI:** 10.3389/fnhum.2013.00826

**Published:** 2013-12-10

**Authors:** Hidenori Aizawa, Wanpeng Cui, Kohichi Tanaka, Hitoshi Okamoto

**Affiliations:** ^1^Department of Molecular Neuroscience, Medical Research Institute, Tokyo Medical and Dental UniversityBunkyo-ku, Tokyo, Japan; ^2^Core Research for Evolutional Science and Technology, Japan Science and Technology AgencyChiyoda-ku, Tokyo, Japan; ^3^Laboratory for Developmental Gene Regulation, RIKEN Brain Science InstituteWako, Saitama, Japan

**Keywords:** habenula, depression, monoamines, rapid eye movement sleep (REMS), glutamate transporters, glutamates

## Abstract

Depression occurs frequently with sleep disturbance such as insomnia. Sleep in depression is associated with disinhibition of the rapid eye movement (REM) sleep. Despite the coincidence of the depression and sleep disturbance, neural substrate for depressive behaviors and sleep regulation remains unknown. Habenula is an epithalamic structure regulating the activities of monoaminergic neurons in the brain stem. Since the imaging studies showed blood flow increase in the habenula of depressive patients, hyperactivation of the habenula has been implicated in the pathophysiology of the depression. Recent electrophysiological studies reported a novel role of the habenular structure in regulation of REM sleep. In this article, we propose possible cellular mechanisms which could elicit the hyperactivation of the habenular neurons and a hypothesis that dysfunction in the habenular circuit causes the behavioral and sleep disturbance in depression. Analysis of the animals with hyperactivated habenula would open the door to understand roles of the habenula in the heterogeneous symptoms such as reduced motor behavior and altered REM sleep in depression.

## Introduction

Depression is a pervasive disorder with a combination of symptoms such as reduced motivation, lack of pleasure and insomnia. The heterogeneity of these symptoms makes it difficult to elucidate the molecular mechanism for the pathophysiology in depression.

Lateral habenula (LHb) is an evolutionarily conserved structure (Aizawa et al., 2011) and has long been known as a nucleus which negatively regulates the monoaminergic systems in the central nervous system, since stimulation of LHb inhibits the firing activity of serotonergic (Wang and Aghajanian, 1977) and dopaminergic neurons (Christoph et al., 1986; Matsumoto and Hikosaka, 2007) in the brain stem. Strategic position of LHb in regulation of the monoamines, such as serotonin and dopamine, prompted researchers to hypothesize a role of LHb in psychiatric disorders such as depression (Hikosaka, 2010).

More recently, increasing numbers of animal and human imaging studies unraveled the evidence for the altered activity of LHb is associated with depressive symptoms such as behavioral despair (Yang et al., 2008; Li et al., 2011), lack of pleasure (Li et al., 2013) and sleep disturbance (Aizawa et al., 2013).

In this review, we discuss possible mechanisms by which the lateral habenular neurons result in pathological activation and propose a hypothesis that hyperactivated habenula could lead to the heterogeneous symptoms observed in depression.

## Changes in the habenular activity in depression

Stress is one of the prominent factors predisposing the depressive symptoms and has been used to induce the animal model for depression. Acute and chronic inescapable stress induce the reduced motor activity in a given situation and elicits the depression-like behaviors such as immobility under tail suspension, reduced motivation to escape from aversive condition (Maier, 1984; Henn and Vollmayr, 2005).

In the animal models for depression, the changes in brain activity associated with depressive behaviors were investigated by 2-deoxyglucose utility (Caldecott-Hazard et al., 1988) or cytochrome oxidase activity (Shumake et al., 2003). These studies consistently found that LHb showed higher metabolic rate in depressed animals than control suggesting a role of hyperactivation of LHb in depression. Analysis of helpless rat provided further insight into understanding the cellular basis of hyperactivation of LHb. More recently, Li et al. (2011) found that excitatory synapse in LHb of the helpless rat was potentiated with an enhanced presynaptic release probability. These results suggested that increased presynaptic action onto LHb neurons contributes to the symptoms observed in the animal model of depression.

On the other hand, it remained unclear until recently how the neurons in LHb act when the animals perceived the unpleasant condition. A series of electrophysiological studies using behaving monkey unraveled LHb neurons acting in an opposite way to the midbrain dopaminergic neurons when the animal experienced the aversive stimuli or absence of the conditioned stimuli predicting reward (Matsumoto and Hikosaka, 2007, 2009). Since impairment of dopaminergic transmission results in suppressed motor behavior (Wise, 2004), it is proposed that repetitive stress as aversive stimuli sensitize LHb neurons to increase the baseline activity, which may lead to the continuous reduction of the firing activity in the midbrain dopaminergic neurons as well as motor behavior (Hikosaka, 2010).

In accordance with findings in the animal models, human imaging studies reported that tryptophan-depletion treatment, which usually deteriorates the symptoms in depressive patients, increased the cerebral blood flow in the habenular region (Morris et al., 1999; Roiser et al., 2009). Structural changes in the habenula of patients with depression were also detected (Ranft et al., 2010; Savitz et al., 2011), supporting the hypothesis that hyperactivated habenula underlies the pathophysiology of depression. Accordingly, it is proposed that deep brain stimulation (DBS) to suppress the neural activity in LHb could be a novel treatment for treatment-resistant depression (TRD; Sartorius and Henn, 2007; Hauptman et al., 2008). Indeed, first trial for treatment of TRD by DBS to LHb induced remission of the depressive symptoms (Sartorius et al., 2010).

Taken together, accumulating evidences from the animal and human studies suggested that hyperactivated LHb is associated with symptoms in depression.

## Cellular mechanism for the hyperactivation of the habenula

**Figure 1:**
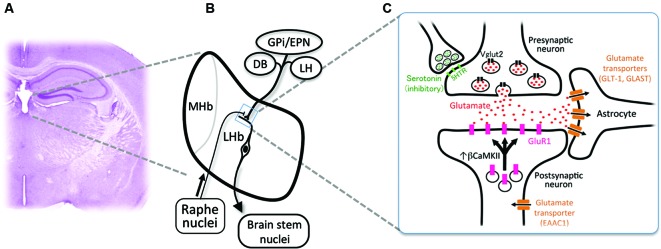
**Cellular mechanism for the excessive excitability in the lateral habenular neurons. (A)** Coronal section of the adult mouse brain showing the anatomical position of the medial and lateral habenulae by Nissl staining. **(B)** Schematic diagram showing the orientation of the MHb and LHb receiving the inputs from GPi/EPN, DB and LH and projecting to the brain stem nuclei. LHb neurons also receive ascending afferent fibers from the serotonergic raphe nuclei. **(C)** Schematic diagram showing molecules essential in the glutamatergic synaptic transmission. Glutamate (red dots) is transported into the synaptic vesicle at the axonal terminal of presynaptic neurons by vesicular glutamate transporter 2 (black rectangles, Vglut2). Serotonin (green dots) acts on the presynaptic axonal terminal through serotonin receptors (light green rectangles, 5-hydroxytryptamine (serotonin) receptor (5HTR)) to inhibit the excitatory transmission. Released glutamate binds and activates the a-amino-3-hydroxy-5-methyl-4-isoxazole propionic acid (AMPA)-type glutamate receptor containing GluR1 subunit (pink rectangles) whose recruitment to the synapse is regulated by β form of calcium/calmodulin-dependent kinase II (*β*CaMKII). Glutamate transporters (brown rectangles) expressed in astrocytes (GLT-1 and GLAST) and neurons (EAAC1) clear the glutamate released to synaptic cleft.

Habenula consists of the medial habenula (MHb) and LHb, each of which has distinct neural connectivity and gene expression. Specifically, LHb receives inputs from diverse structures such as internal segment of globus pallidus/entopeduncular nucleus (GPi/EPN), diagonal band (DB) and lateral hypothalamus (LH), and projects primarily to the brain stem nuclei (Figures 1A, B; Herkenham and Nauta, 1977, 1979). Rat habenula is further divided into more than 10 subnuclei expressing specific sets of genes (Andres et al., 1999; Aizawa et al., 2012). Despite the heterogeneity of neural connectivity and gene expression of the habenular subnuclei, majority of the medial and lateral habenular neurons use glutamate as neurotransmitter (Geisler et al., 2007; Aizawa et al., 2012).

LHb expresses presynaptic and postsynaptic markers for glutamatergic transmission in mouse. Axonal terminals in LHb express presynaptic marker protein vesicular glutamate transporter 2 (Sakata-Haga et al., 2001; black rectangles in Figure 1C). It is reported that glutamatergic synaptic transmission is mediated by AMPA-type glutamate receptor expressed in LHb (Petralia and Wenthold, 1992; Li et al., 2011; Shabel et al., 2012; pink rectangles in Figure 1C). A subtype of AMPA-type glutamate receptor is known to be mobilized with recycling endosomes to the synapse upon neural excitation (Malinow and Malenka, 2002). Considering a crucial role of synaptic mobilization of GluR1 in synaptic plasticity (Malinow and Malenka, 2002), it is reasonable to consider the possibility that neuronal activity in LHb is controlled by insertion of GluR1 molecule into synapses. This is proved recently by identifying *β*CaMKII as a regulator of LHb neuron function (Li et al., 2013). Congenital helpless rat showed up-regulation of *β*CaMKII in LHb, and knockdown of *β*CaMKII mRNA via RNA interference ameliorated the depressive symptoms. Since up-regulation of *β*CaMKII increases the synaptic expression and delivery of glutamate receptor GluR1 (Groth et al., 2011), these results indicated that *β*CaMKII-GluR1 pathway determines the excitability of LHb neurons. Considering the excitatory response of LHb neurons to aversive stimuli, it is suggested that repetitive stress may sensitize the synapse in LHb by unknown mechanism to induce prolonged activation of LHb, which, in turn, causes the up-regulation of *β*CaMKII-GluRI pathway at the onset of depression (Hikosaka, 2010; Li et al., 2013).

Glutamate released from the presynaptic axonal terminal plays central role in excitability of the synapse. Glutamate transporters expressed in neurons (EAAC1) and astrocytes (GLT-1 and GLAST) clear the glutamate released into the synapse (brown rectangles in Figure 1C). Blockade of glutamate transporter activity prolongs the response of the excitatory synapse by elongating the decay of excitatory postsynaptic current (Tzingounis and Wadiche, 2007). Among three transporters, GLT-1 is the quantitatively dominating glutamate transporter, since more than 90% of the transport activity in forebrain tissue extracts disappeared by immunoprecipitation by antibody against GLT-1 (Haugeto et al., 1996). In support of this view, mice lacking GLT-1 showed elevation of the glutamate concentration in the synaptic cleft for longer periods and died of lethal seizure (Tanaka et al., 1997; Mitani and Tanaka, 2003). Thus, it is likely that alteration in the glial glutamate transporter activity plays a role in determining excitability in the habenula.

**Figure 2:**
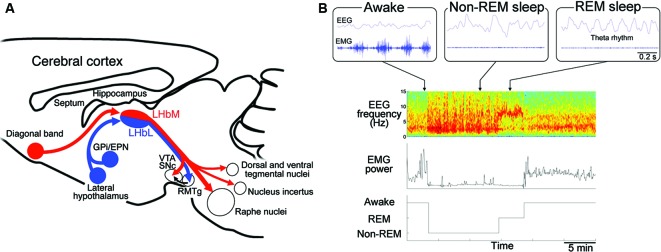
**Regulation of the rapid eye movement (REM) sleep by habenular projection. (A)** Efferent targets of the LHb in regulation of REM sleep. Schematic diagram of a sagittal section of the mouse brain showing the afferent and efferent connectivity of LHbM (red) and LHbL (blue). LHbM receives inputs preferentially from the DB and send the axons to the serotonergic raphe nuclei, dorsal and ventral tegmental nuclei containing GABAergic neurons, nucleus incertus producing the neuropeptide relaxin-3 and dopaminergic ventral tegmental area (VTA) and substantia nigra, pars compacta (SNc). On the other hand, LHbL receives inputs preferentially from GPi/EPN and LH and sends the axons to the GABAergic rostromedial tegmental nucleus (RMTg). **(B)** Categorization of the sleep stage into awake, REM sleep and non-REM sleep according to the electroencephalogram (EEG) and electromyogram (EMG). Blue traces in the upper three panels show raw activity in EEG (top) and EMG (bottom) during the awake (left), non-REM sleep (middle) and REM sleep (right). Lower panels show an example of classification of the sleep record based on the spectrogram of EEG (top), EMG power (middle) into the three stages (bottom). Spectrogram represents a pseudocolor plot of the power of each frequency range for each 4 s window. Note that the power in the delta (1–3 Hz) and theta (5–8 Hz) band dominates with reduced EMG power during non-REM and REM sleep period, respectively. In the awake state, high EMG power is evident.

Excitability of neurons is also under the influence of inhibitory GABAergic inputs in LHb expressing GABA_A_ (Pirker et al., 2000) and GABA_B_ receptors (Margeta-Mitrovic et al., 1999). The afferents to LHb originate primarily from the GPi/EPN as well as LH and DB (Herkenham and Nauta, 1977). LHb consists of medial (LHbM, red in Figure 2A) and lateral division (LHbL, blue in Figure 2A), each of which receives afferents preferentially from DB and GPi/EPN, respectively (Herkenham and Nauta, 1977). Although previous reports showed many of the afferents from GPi/EPN were GABAergic (Araki et al., 1984), recent studies revealed that GPi/EPN projecting to LHb consisted of GABAergic and glutamatergic neurons (Shabel et al., 2012). On the other hand, electrical stimulation of the incoming fibers produced brief hyperpolarizing postsynaptic potential in LHbM neurons (Chang and Kim, 2004). Interestingly, this brief hyperpolarization elicited a persistent depolarization of habenular neurons and promotes long-lasting discharges in majority of the cells in LHbM. These facts imply that LHb neurons may increase excitability via prolonged hyperpolarized state due to the brief inhibitory inputs, although the net effect of excitatory and inhibitory inputs to the synapse determines the excitability of the postsynaptic neurons in general. Inhibitory neurons expressing marker gene for GABAergic neurons were found in LHb, although a role of these neurons in modulation of the other excitatory projection neuron in LHb remains unclear (Brinschwitz et al., 2010).

LHb also receives innervation by the dopaminergic (Phillipson and Griffith, 1980; Aizawa et al., 2012) and serotonergic fibers (Morin and Meyer-Bernstein, 1999; Leger et al., 2001; de Jong et al., 2010) originated from the brain stem (Figure 1B). In addition, LHb neurons express specific subtype of receptors for monoamines such as dopamine type 2 receptor (Weiner et al., 1991; Aizawa et al., 2012) and serotonin 2c receptors (Mengod et al., 1990; Aizawa et al., 2012). A recent study reported that serotonin had suppressive effect on excitatory input from GPi/EPN to LHb neurons presynaptically (Shabel et al., 2012; green dots in Figures 1B, C). Consistent with this, functional imaging studies using positron emission tomography (Morris et al., 1999) and functional magnetic resonance imaging (Roiser et al., 2009) showed that reduction of serotonin metabolism by tryptophan-depletion in patients with depression, increased the cerebral blood flow in the habenula. Thus, taking the inhibitory effect of serotonergic inputs on the LHb excitability into consideration, antidepressant drugs such as serotonin-selective reuptake inhibitors may also act, at least in part, on the presynaptic terminal of axons to suppress the hyperactivation of LHb.

Dopamine also has an excitatory effect on the activity in LHb neurons (Kowski et al., 2009), although the direction of firing rate changes in LHb neurons varied along time course after application of the dopaminergic agonist (Jhou et al., 2013) and depends on the subnucleus. In chronic social defeat stress model of depression, the animals showed an increase in firing rate of the midbrain dopaminergic neurons both *in vitro* (Krishnan et al., 2007) and* in vivo* (Cao et al., 2010). Chronic activation of the dopaminergic inputs to LHb might contribute to the hyperactivation of the habenula in depression.

Although a recent study unravels that recruitment of glutamate receptor via upregulated *β*CaMKII signaling causes hyperactivation of LHb neurons associated with depressive symptoms (Li et al., 2013), it remained elusive how neurons in LHb start being activated to increase *β*CaMKII. As we discussed above, altered function of the genes expressed either in presynaptic and postsynaptic neurons or glial cells could account for the hyperactivation of LHb neurons. Thus, examining *β*CaMKII activity and the depressive behaviors in the mutant animals lacking these genes would enable us to identify the upstream molecules which act on the *β*CaMKII-GluR1 pathway.

## Role of the habenula in sleep

Mammalian sleep consists of REM sleep and non-REM sleep. It is repeatedly reported that patients with depression show shortened latency to the onset of REM sleep, longer duration of REM sleep and increased eye movement frequency during REM sleep (Seifritz, 2001). Serotonin has been implicated as a molecule playing an critical role in transition between non-REM sleep and REM sleep (Jouvet, 1969). Antidepressants such as imipramine (Vogel et al., 1975) and serotonin-selective reuptake inhibitor, fluoxetine (Slater et al., 1978) reduce REM sleep period, suggesting close association between depressive symptoms and sleep disturbance. These findings suggest that alteration of REM sleep could be an endophenotype of depression (Hasler et al., 2004; Gottesmann and Gottesman, 2007; Modell and Lauer, 2007; Steiger and Kimura, 2010). Furthermore, identifying the neural substrate for altered REM sleep in depression must be useful not only for understanding the pathophysiology of depression but also for developing novel diagnostic and therapeutic strategies. In rodents, REM sleep period is identified by appearance of 5–8 Hz theta rhythm in EEG originated primarily from septohippocampal activity with muscle atonia (Figure 2B).

Considering the regulatory role of LHb in serotonergic system, it is reasonable to conceive the idea that LHb is involved in regulation of sleep. Indeed, previous studies showed lesion of the fasciculus retroflexus (FR), which affects the efferent projections from MHb and LHb as well as axons originating from the basal forebrain and passing through the habenular region, led to the fragmentation of the REM sleep bouts (Haun et al., 1992; Valjakka et al., 1998).

However, it remained unclear whether LHb plays a role in regulation of REM sleep. We recently addressed this by examining the REM sleep based on electrophysiological recording in the rats with specific lesion in LHb (Aizawa et al., 2013). Results showed that LHb lesion reduced the length of REM sleep period by shortening the single REM sleep bout in the rats, suggesting that firing activity of LHb neurons may be indispensable for maintenance of REM sleep. In line with this, recent electrophysiological studies found that LHb neurons fire synchronously in a frequency of theta range with close temporal association with hippocampal theta rhythm during REM sleep (Aizawa et al., 2013; Goutagny et al., 2013). These results indicated that synchronous activity in LHb is essential for the maintenance of REM sleep via modulation of serotonergic activity.

For efferent projection, neurons in LHbM (red in Figure 2A) preferentially projected to the serotonergic raphe nuclei than those in LHbL (blue in Figure 2A). Consistent with this, single cell labeling experiments showed that the synchronous firing was more frequently observed in LHbM neurons than in LHbL. Furthermore, the inhibitory effect of LHb on REM sleep depended on the intact serotonergic activity in the median raphe (Aizawa et al., 2013). These results suggested that LHb regulates REM sleep via serotonergic neurons in the median raphe.

It is interesting that many of the tegmental nuclei receiving the LHb efferent projection are implicated in REM sleep regulation. For example, nucleus incertus in the tegmentum contain neurons which produce neuropeptide relaxin-3, whose infusion into the rat septum elicits the theta oscillation characteristic to REM sleep (Ma et al., 2009; Figure 2A). Dorsal and ventral tegmental nuclei, both of which contain a large population of GABAergic neurons, are also activated during REM sleep (Bassant and Poindessous-Jazat, 2001; Kocsis et al., 2001; Bassant and Poindessous-Jazat, 2002; Torterolo et al., 2002; Figure 2A). Examining the activity changes in these structures in the LHb-lesioned animals will clarify their roles in REM sleep.

Taken together, it is suggested that LHb neurons change their firing pattern when the animals are in REM sleep to maintain its stability. Considering the inhibitory effect of LHb lesion on REM sleep stability, it is likely that hyperactivated LHb up-regulates REM sleep.

## Perspectives

According to the evidence obtained so far, it is hypothesized that hyperactivated LHb causes heterogeneous symptoms such as reduced motor behavior and altered REM sleep. This hypothesis will be addressed more directly by future study which examines whether the animals with hyperactivation of the LHb show depressive behaviors and sleep disturbance with up-regulation of REM sleep.

## Conflict of interest statement

The authors declare that the research was conducted in the absence of any commercial or financial relationships that could be construed as a potential conflict of interest.
